# Our Next Volume

**Published:** 1877-12-20

**Authors:** 


Our Next Volume.—We are pleased to inform our readers that our next volume, commencing with the January issue, will be gotten out in better type, and will contain more reading matter than the volume now closing. The present low price of subscription will be continued.

